# Evaluation of IVIM and FACT imaging for early detection of rotator cuff muscle degeneration and physiotherapy exercise effects

**DOI:** 10.3389/fspor.2025.1688162

**Published:** 2025-10-30

**Authors:** Ling Teng, Mingru Huang, Hongyue Tao, Xiao Zhang, Sijia Feng, Xiner Du, Zhiwei Qin, Xiangwen Li, Yiwen Hu, Changyan Liu, Yuxue Xie, Shuang Chen

**Affiliations:** ^1^ Department of Radiology, Institute of Medical Functional and Molecular Imaging, Huashan Hospital, Fudan University, Shanghai, China; ^2^ Department of Sports Medicine, Sports Medicine Institute of Fudan University, Huashan Hospital, Fudan University, Shanghai, China; ^3^ MR Collaboration Innovation Department, Shanghai United Imaging Healthcare Co., Ltd., Shanghai, China; ^4^ National Clinical Research Center for Aging and Medicine, Huashan Hospital, Fudan University, Shanghai, China

**Keywords:** magnetic resonance imaging, shoulder, rotator cuff, muscle, IVIM

## Abstract

**Background:**

This study investigated novel magnetic resonance imaging (MRI) techniques, including intravoxel incoherent motion (IVIM) and fat analysis & calculation technique (FACT) to detect early pathologic changes in rotator cuff (RC) muscle in patients with shoulder pain and evaluate rehabilitation effects.

**Methods:**

MRI examinations, including IVIM and FACT, and clinical scores were collected in patients with unilateral shoulder pain at baseline (*n* = 48) and 3-month follow-up (*n* = 42) after physiotherapy exercise. The contralateral shoulder served as control. IVIM-derived parameters (apparent diffusion coefficient, ADC; diffusion coefficient, D; pseudo-diffusion coefficient, D*; perfusion fraction, f; and their product, fD*) and FACT-derived parameters (fat fraction, FF) were measured in four RC muscles. Clinical assessment included Constant Score (CS), the American Shoulder and Elbow Surgeons (ASES), and the Visual Analog Scale (VAS). Differences in imaging parameters were compared between the affected and the contralateral shoulder. Changes in imaging parameters and clinical scores after exercise rehabilitation were analyzed between baseline and 3-month follow-up. Correlation analysis was conducted between the change in imaging values and the change in clinical scores.

**Results:**

At baseline, D* values of the affected sides in four RC muscles were lower compared to the contralateral sides (*p* < 0.05). f and fD* values of the affected sides were higher and lower respectively, compared to the contralateral sides and showed a difference in infraspinatus and subscapularis (*p* = 0.03 and *p* = 0.04). No significant differences for FF and other IVIM-derived parameters in either muscle were observed. After the physiotherapy exercise, all of the clinical scores showed significant improvements compared to the baseline (all *p* < 0.05). Regarding imaging parameters, only D* values showed a significant increase in the supraspinatus muscle (*p* < 0.05). Additionally, the change in D* and f correlated with CS, ASES scores and VAS scores respectively. FF showed a decreasing trend but lacking significance (*p* > 0.05).

**Conclusion:**

Capillary flow impairment (reflected by D*) appears more prominent than fat infiltration (reflected by FF) in the early RC muscle degeneration. IVIM-derived parameters, particularly D* may assist clinicians in detecting early-stage muscle degeneration and provide a non-invasive method for monitoring rehabilitation outcomes.

## Introduction

Shoulder pain, commonly caused by rotator cuff (RC) disorders, is a frequent musculoskeletal complaint (1, 2). Physiotherapy is widely used to manage these disorders, aiming to reduce pain, improve range of motion (ROM), and restore function (3).

Muscle quality is a critical determinant of joint function (4). Individuals with RC disorders have demonstrated degeneration of RC muscles, including inflammation, atrophy, fibrosis, and fatty infiltration (FI), which are often considered irreversible (4–7). Magnetic resonance imaging (MRI) is one of the most useful tools for evaluating muscle conditions due to its superior soft-tissue resolution and multiparameter imaging characteristics (8–10). Although significant research has focused on muscle atrophy and FI after arthroscopic rotator cuff repair (ARCR), particularly in relation to tendon integrity (5, 11–16), most studies address advanced stages of RC tears. There remains a significant gap regarding early-stage muscle degeneration in individuals before progressing into irreversible shoulder joint disorders, such as RC tears. Meanwhile, detecting such early changes is essential for optimizing physiotherapy and guiding effective treatment strategies to prevent further deterioration.

One promising approach for muscle assessment is the evaluation of free water diffusion using intra-voxel incoherent motion (IVIM) imaging (17). IVIM uses multi-b-value diffusion-weighted imaging (DWI) to simultaneously evaluate both the true diffusion of tissue water and the microvascular blood water (so-called pseudo-diffusion) in skeletal muscles (18–23). IVIM-derived parameters, apparent diffusion coefficient, ADC, diffusion coefficient, D, are closely related to the ability of water molecules to diffuse freely with or without the influence of blood flow perfusion; pseudo-diffusion coefficient, D*, perfusion fraction, f, are proven to be related to capillary circulation; and their product, fD*, is associated with tissue perfusion rate (22, 24, 25). While IVIM has been used to study muscle perfusion in healthy individuals under various conditions (26–31), its application in pathological muscle states remains limited, primarily to inflammatory myopathies (25, 32–34). Given evidence that treadmill training can enhance limb perfusion (35), we hypothesize that IVIM may also detect perfusion improvements in RC muscles following physiotherapy.

For evaluating fat infiltration (FI), the 3D Fat Analysis & Calculation Technique (FACT) is an emerging MRI sequence that provides precise water-fat separation and volumetric fat quantification. Compared to Dixon-based techniques, FACT offers improved accuracy in regions with complex fat-water interactions due to its robustness against magnetic field inhomogeneity (36). While FACT has shown promise in evaluating fat content, particularly in bone marrow (37), its application to assessing FI in muscle tissue remains underexplored. Previous studies have demonstrated that inflamed muscles exhibit elevated diffusivity, while fat-infiltrated muscles show reduced diffusivity, primarily in the context of myopathies (38). We supposed that IVIM could reveal more RC muscle abnormalities before they become evident at conventional morphologic T1-, T2-, or FACT MR sequences.

Therefore, the purpose of this study is to (1) use IVIM and FACT to detect early pathological changes in RC muscles condition in patients with shoulder pain by comparing the affected shoulders with the contralateral shoulders; (2) use IVIM and FACT to assess changes in RC muscles condition after physiotherapy exercise; (3) investigate the correlations between IVIM-, FACT- derived parameters and clinical outcomes in patients after physiotherapy exercise.

## Methods and materials

### Study population

**Figure 1:**
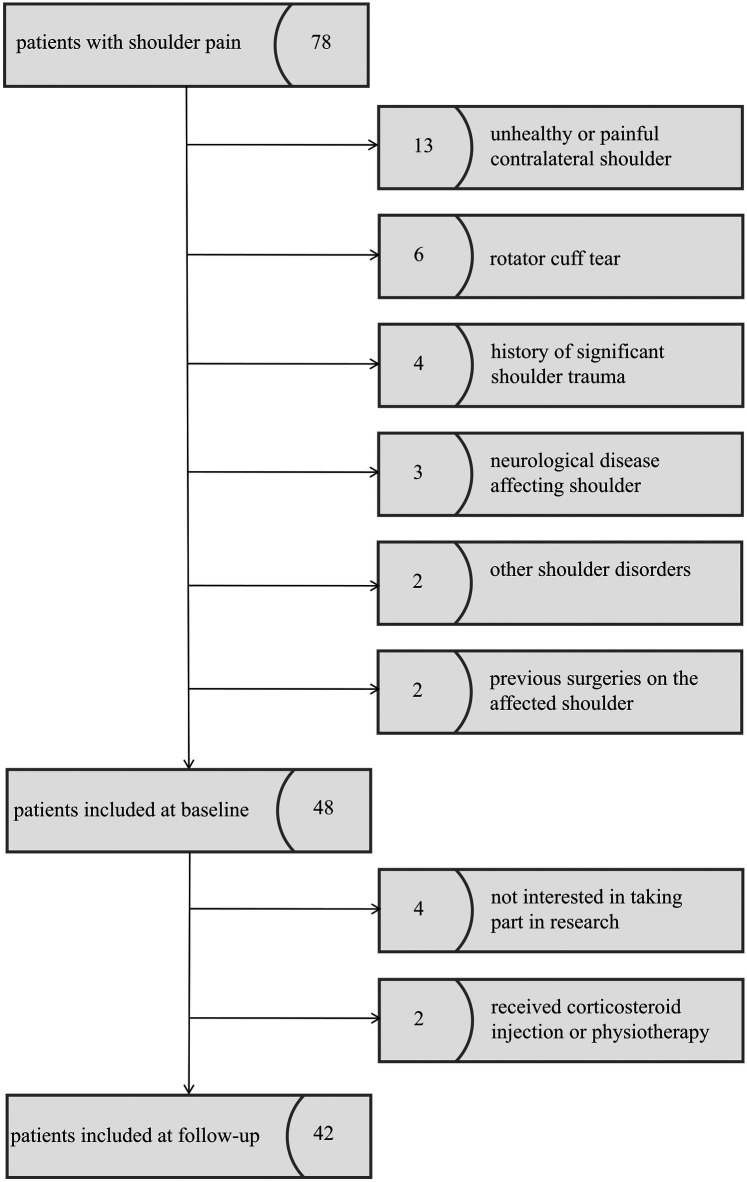
Flowchart of study patients.

This prospective study was approved by the Health Sciences Institutional Review Board of our hospital. Informed consent was obtained from all patients. From June 2023 to April 2025, patients that had a diagnosis of shoulder pain attributable to a rotator cuff disorder (e.g., cuff tendonitis, impingement syndrome, tendinopathy) were enrolled. If no exclusion criteria were applied, patients were invited for a single follow-up examination at 3 months after physiotherapy exercise. The inclusion criteria were as follows:(1) unilateral shoulder pain, (2) the contralateral shoulder reported no function loss and pain-free, and (3) body mass index (BMI ≤ 30 kg/m^2^). The exclusion criteria included: (1) rotator cuff tear (e.g., partial tear or full-thickness tear), (2) significant shoulder trauma (e.g., dislocation or fracture), (3) neurological disease affecting the shoulder, (4) other shoulder conditions (e.g., inflammatory arthritis, frozen shoulder, or glenohumeral joint instability), (5) previous surgeries on either the affected or contralateral shoulder, and (6) received corticosteroid injection or physiotherapy for shoulder pain during physiotherapy exercise (Figure 1).

### MRI examination protocol

The MRI examinations of patients were conducted at baseline (bilateral shoulder), and at 3-month follow-up (affected shoulder) after physiotherapy exercise. Lifting or rehabilitation exercises were prohibited for at least 2 h before MRI scans to avoid activity-related biochemical effects. All MRI examinations were performed using a 3.0-T (uMR 790, United Imaging Healthcare, Shanghai, China) MRI scanner with an 8-channel phased array shoulder coil. Standard clinical MRI protocols, including axial and sagittal-oblique proton density fast spin-echo fat-saturated sequence (PDWI) and coronal-oblique fat-saturated T2-weighted sequence (T2WI).

IVIM adopted the following parameters: TR/TE, 4000/61.7 ms; bandwidth, 1,280 Hz/pixel; field of view, 16 × 16 cm; slice thickness, 4.0 mm; slices, 25; and sixteen b values, 0, 10, 20, 40, 80, 110, 140, 170, 200, 300, 400, 500, 600, 700, 800 and 900 s/mm^2^. And FACT adopted the following parameters: TR/TE, 12.23/2.47 ms; flip angle, 3°; bandwidth, 930 Hz/pixel; field of view, 16 × 16 cm; slice thickness, 1.4 mm; slices, 40. The slice thickness and scanning range of all the images were consistent.

### Image analyses

**Figure 2:**
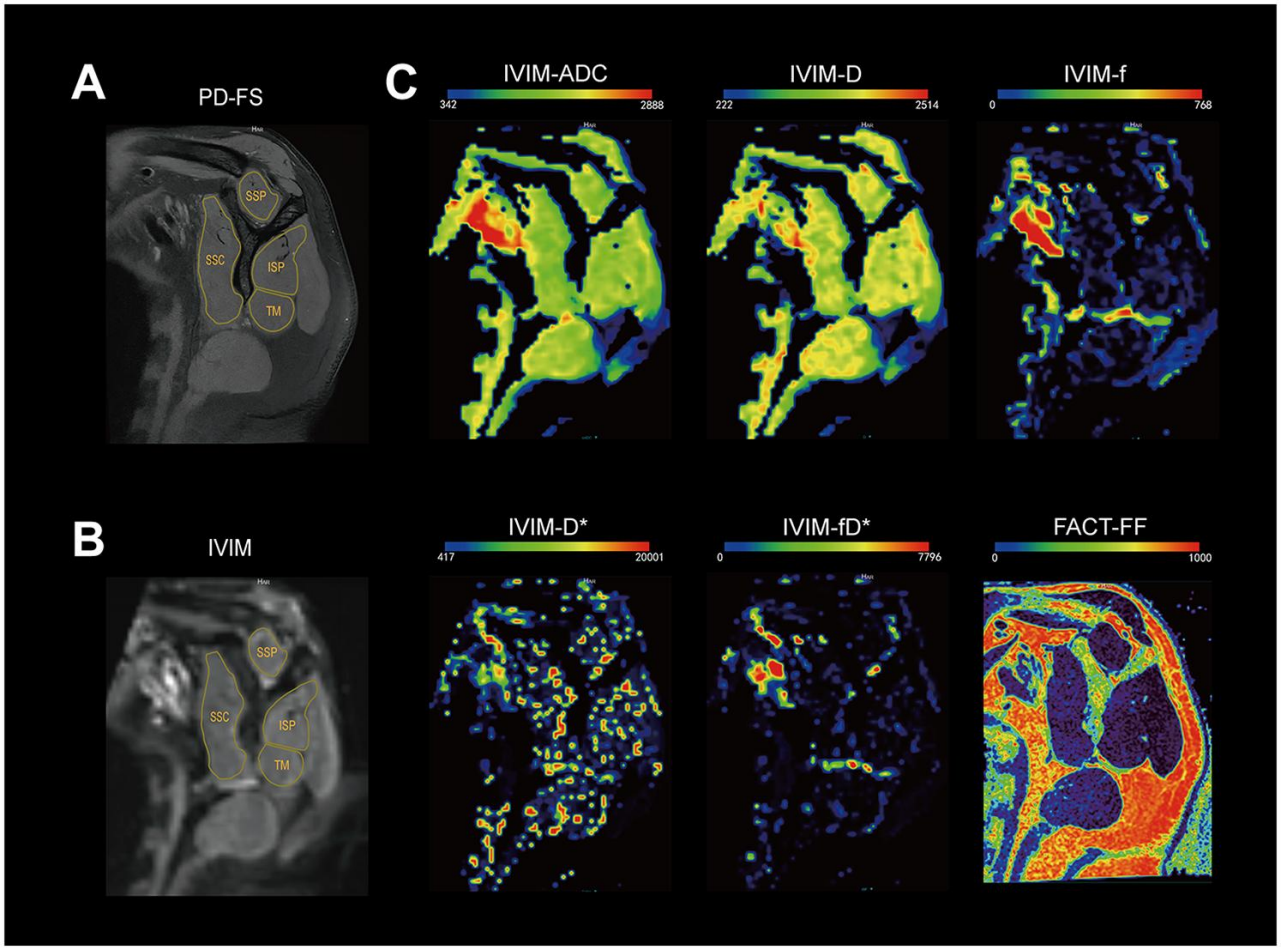
The representative slice of the Y-shaped view for rotator cuff muscles. **(A)** images for proton density fat-saturated; **(B)** raw intravoxel incoherent motion (IVIM) b0 image with region of interest outlined for supraspinatus (SSP), infraspinatus (ISP), subscapularis (SSC), and teres minor(TM); **(C)** Parameter maps of IVIM (ADC, D, f, D*, fD*) and FF map for FACT. The color-bar unit is 10^−6^ mm^2^/s, 10^−6^ mm^2^/s, 10^−3^, 10^−5^ mm^2^/s, 10^−5^ mm^2^/s, 10^−3^, respectively.

Conventional sequences including PDWI and T2WI provided initial clinical diagnosis of tendinosis, partial- or full-thickness tears, and other structural abnormalities. IVIM and FACT data were imported into the post-processed workstation (uWS-MR Advanced Postprocess Workstation, United Imaging Healthcare, Shanghai, China) to obtain parametric maps as well as parameters for IVIM (apparent diffusion coefficient, ADC; diffusion coefficient, D; pseudo-diffusion coefficient, D*; perfusion fraction, f; and their product, fD*) and parameters for FACT (fat fraction, FF). The regions of interest (ROI) were manually drawn around the four RC muscles (supraspinatus, SSP; infraspinatus, ISP; subscapularis, SSC; and teres minor, TM) at the Y-shaped view (Figure 2B) to obtain values of each parameter. Attention was given to excluding areas influenced by bones, fasciae, fat, and large vessels. To ensure consistency, these ROIs were then precisely copied and applied to the exactly matched slices on the co-registered IVIM and FACT parametric maps using the image analysis software's co-registration and ROI propagation tools. This method guaranteed that all parameters were measured from identical anatomical locations across all sequences. Furthermore, the PDWI sequence, due to its excellent depiction of musculoskeletal anatomy, was used as the anatomical reference for manual delineation of ROIs (Figure 2A). Two observers conducted the measurement to evaluate the inter-reader reproducibility. Observer 1: An attending physician specializing in shoulder disorders with ten years of clinical experience. Observer 2: A graduate student in radiology with three years of focused research experience on shoulder imaging. Both observers were trained on the standardized measurement protocol prior to the study to ensure consistency.

### Physiotherapy exercise

The rehabilitation protocol for patients was created by fellowshipped sports medicine physicians with 20 years of experience and rehabilitation physicians of our hospital, which comprised 13 movements including improvements in shoulder joint range of motion, scapular control, and trunk stability, summarized and improved from previous studies (39–41). Details were shown in Supplementary Material Table S1. Patients were given a rehabilitation booklet with instructions on their exercise program and were advised to perform at least three sets per week. The online check-in mini-program was established to monitor patients' physiotherapy exercise progress. Patients record their attendance after each rehabilitation session. All exercises are conducted strictly within the patient's pain-free range of motion to ensure safety and effectiveness.

### Clinical assessments

The clinical evaluations were conducted at baseline and 3-month follow-up (affected shoulder) after physiotherapy exercise, including the Constant and the American Shoulder and Elbow Surgeons (ASES) (42). Additionally, the visual analog scale (VAS) for pain was used to register the worst pain felt during ordinary activities over 24 h. In the VAS scoring system, 10 indicates the worst pain, while 0 indicates no pain.

### Statistical analysis

SPSS 25.0 software (IBM, Chicago, IL, USA) was used for the analysis. The paired Student's *t*-test or Wilcoxon matched-pairs signed rank test was used to analyze the difference in data between the affected and contralateral shoulders and to compare the imaging and clinical data of affected shoulders between the baseline and follow-up after physiotherapy exercise. Differences in imaging parameters among the healthy side and the affected side of different time points were compared using one-way analysis of variance and multiple comparisons. Spearman correlation coefficient was used to analyze the correlations between the change in imaging values and the change in clinical scores. The differences corresponding to *p* < 0.05 are considered statistically significant. Intraclass correlation coefficients (ICCs) were calculated to assess the measurement reproducibility across observers. The ICCs were graded as follows: excellent reliability, 0.75 ≤ ICC ≤ 1; fair to good, 0.4 ≤ ICC ≤ 0.75; and poor, 0 ≤ ICC ≤ 0.4).

## Results

Patient characteristics are shown in Table 1. 48 patients were analyzed at baseline. 42 patients completed 3-month follow-up examinations after physiotherapy exercise. There were no significant differences in patients' general characteristics between baseline and follow-up. The average rehabilitation exercise regimen is 5.5 (range, 3–18) per week. The ICC values were 0.76 for ADC, 0.79 for D, 0.82 for D*, 0.86 for f, 0.70 for fD*, and 0.83 for FF.

**Table 1:** Demographic data of the patient with shoulder Pain^a^.

Characteristic	Baseline	Follow-up
Patients	48	42
Age, year	33.72 ± 9.67	35.30 ± 11.93
Body mass index, kg/m^2^	22.24 ± 3.76	21.58 ± 2.91
Male: Female	20:28	17:25
Symptom duration, months	10 (26)	10 (14.5)
Affected shoulder (left: right)	21:27	20:22
Hand dominance (left: right)	2:46	1:41

^a^ Data was presented as mean ± standard deviation or median (interquartile range, IQR).

### Comparisons of imaging parameters between the affected and contralateral shoulders at baseline

Results of comparing imaging parameters between the affected and contralateral shoulders at baseline are illustrated in Table 2.

**Table 2:** Comparisons of imaging parameters between the affected and contralateral shoulders in patients with shoulder Pain^a^

Parameter		Affected side	Contralateral side	*P*
IVIM-ADC (×10^−6^ mm^2^/sec)	Supraspinatus	1536.92 ± 95.55	1547.49 ± 75.70	0.41
	Subscapularis	1552.14 (111.39)	1542.86 (92.04)	0.76
	Infraspinatus	1545.90 ± 82.69	1545.15 (108.01)	0.94
	Teres minor	1541.45 ± 122.28	1521.52 (115.42)	0.86
IVIM-D (×10^−6^ mm^2^/sec)	Supraspinatus	1511.95 ± 111.15	1515.90 (82.90)	0.24
	Subscapularis	1473.07 (110.47)	1463.98 (114.32)	0.41
	Infraspinatus	1494.63 ± 84.52	1515.90 (110.20)	0.23
	Teres minor	1476.50 ± 123.85	1476.66 ± 118.94	0.99
IVIM-f (×10^−3^)	Supraspinatus	55.59 ± 21.96	51.82 (29.73)	0.13
	Subscapularis	92.78 ± 33.38	86.99 ± 34.99	0.35
	Infraspinatus	73.67 (35.74)	64.49 ± 23.75	**0**.**03**
	Teres minor	80.81 (47.92)	72.77 (44.15)	0.51
IVIM-D* (×10^−5^ mm^2^/sec)	Supraspinatus	4169.51 ± 1137.75	4690.21 ± 1100.26	**0**.**02**
	Subscapularis	3619.37 ± 1079.69	4145.40 ± 1011.66	**0**.**009**
	Infraspinatus	3745.47 ± 1240.40	4525.98 ± 1300.21	**0**.**002**
	Teres minor	3515.81 ± 1256.56	4058.71 ± 1239.52	**0**.**03**
IVIM-fD* (×10^−5^ mm^2^/sec)	Supraspinatus	237.84 ± 108.50	245.01 (104.39)	0.55
	Subscapularis	315.70 (139.20)	336.56 ± 118.46	**0**.**04**
	Infraspinatus	239.62 (154.46)	303.44 ± 110.22	0.09
	Teres minor	263.58 ± 119.23	299.47 ± 154.30	0.15
FACT-FF(‰)	Supraspinatus	80.65 ± 19.23	76.92 ± 17.90	0.09
	Subscapularis	118.79 ± 27.58	108.80 (27.90)	0.09
	Infraspinatus	90.46 ± 23.94	88.50 (33.70)	0.32
	Teres minor	80.39 ± 19.60	79.53 ± 19.10	0.56

^a^ Data was presented as mean ± standard deviation or median (interquartile range, IQR). *P* values with significant differences are presented in bold.

Within the muscle groups studied, D* values of the affected sides were lower compared to the contralateral sides (all *p* < 0.05). The f values of the affected sides were higher than the contralateral sides in all four muscle groups, with a significant difference in ISP (*p* = 0.03). The fD* values of the affected sides were lower than the contralateral sides in all four muscle groups, with a significant difference in SSC (*p* = 0.04). For other IVIM-derived parameters, values of the affected sides were generally lower than those of the contralateral sides across most muscle groups but lacking significant differences (all *p* > 0.05). Additionally, no significant differences for FF in either muscle group were observed between the affected and contralateral sides though values of the affected sides were generally higher than those of the contralateral sides.

### Post-rehabilitation changes in imaging parameters of the affected shoulders

**Figure 3:**
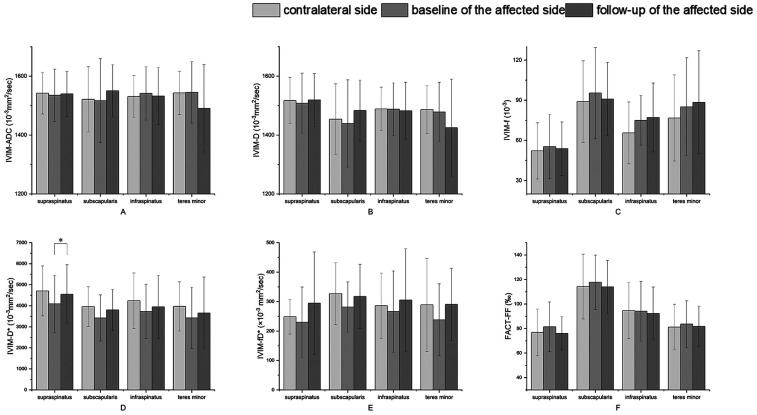
Changes in imaging parameters of the affected shoulders after physiotherapy exercise and comparison of parameters between contralateral shoulders and affected shoulders. **(A–F)** IVIM-derived values, ADC, D, f, D*, fD*, and FACT-derived values, FF. *p < 0.05.

Changes in imaging parameters after exercise of the affected sides are illustrated in Figure 3. In four muscle groups, D* values were increased at follow-up compared to baseline and only showed a significant difference in SSP (*p* = 0.03). However, all values were still lower than those of the contralateral shoulders (*p* > 0.05) (Figure 3D). f values were decreased in SSP and SSC, and increased in ISP and TM at follow-up compared to baseline (*p* > 0.05), and values were all higher than those of the contralateral shoulders (*p* > 0.05). fD* values showed an increased trend compared to the baseline (all *p* > 0.05). D and ADC values at follow-up were slightly increased compared to baseline in SSP and SSC, while in ISP and TM, values showed a decreasing trend (*p* > 0.05). In terms of FF in four muscle groups, values were lower at follow-up compared to baseline but showed no significant difference (*p* > 0.05). Additionally, values were not much different from the contralateral shoulders.

### Post-rehabilitation changes in clinical scores

**Figure 4:**
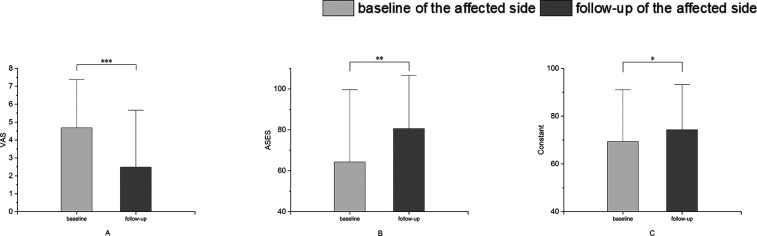
Changes in clinical scores of the affected shoulders over time. (**A**) VAS, visual analog scale for pain score; (**B**) Constant, Constant Score; (**C**) ASES, American Shoulder and Elbow Surgeons score. *p < 0.05. **p < 0.01. ***p < 0.001.

Compared to baseline, the function scores (Constant and ASES) significantly increased (*p* = 0.03 and 0.001), and the VAS pain score markedly decreased at follow-up (*p* < 0.001) (Figure 4). Moreover, nearly all patients (96%) reported remarkable pain relief based on their VAS score, with 4 cases (10%) ranking 0.

### Correlation analysis

There was a weak positive correlation between the change in D* of SSP and the change in Constant, ASES scores (*r* = 0.39, *p* = 0.03; *r* = 0.39, *p* = 0.04, respectively) (Table 3). Moreover, there was a moderate correlation between the change in f and the change in VAS scores (*r* = 0.51, *p* = 0.004). There was no significant correlation shown in other muscle groups.

**Table 3:** Correlations between the change in imaging parameters and the change in clinical scores of SSP^a^.

Clinical score	ADC	D	F	D*	fD*	FF
VAS score						
*r*	−0.01	0.19	0.51	0.33	−0.19	−0.09
*p*	0.95	0.31	**0**.**004**	0.07	0.31	0.62
CS score						
*r*	−0.12	−0.09	0.30	0.39	−0.15	0.03
*p*	0.41	0.65	0.10	**0**.**03**	0.44	0.87
ASES score						
*r*	0.07	0.20	0.31	0.37	0.11	−0.12
*p*	0.70	0.29	0.09	**0**.**04**	0.55	0.53

^a^ Data were calculated by values at 3-month follow-up minus those at baseline. VAS, visual analog scale; CS, constant score; ASES, American shoulder and elbow surgeons. *P* values with significant differences are presented in bold.

## Discussion

This study investigated the effectiveness of IVIM- and FACT-derived parameters in the differentiation of early-stage degeneration from normal muscle and illustrated the correlations between post-rehabilitation muscle changes and clinical outcomes in patients with shoulder pain. The result of our study suggested that the capillary flow reflected by IVIM-derived D* has priority over the FI change reflected by FACT in the early detection of RC muscles degeneration. For muscle condition after physiotherapy exercise, capillary flow showed a significant improvement in SSP. Also, the changes in IVIM-derived D* showed associations with improvements in Constant, ASES scores. Our results may suggest that IVIM may assist clinicians in detecting early-stage muscle degeneration and monitoring the post-rehabilitation treatment effect accurately and non-invasively.

Recently, IVIM studies have mainly reported parameters in healthy individuals, with several studies evaluating pathological conditions that result in changes to skeletal muscle (25). In prior studies, IVIM-derived f, D, D*, and fD* values were reported to as approximately 6%, 1.5 × 10^−3^ mm^2^/s, 12 × 10^−3^ mm^2^/s, and 0.8 × 10^−3^ mm^2^/s, respectively, in the healthy shoulder muscles (28, 29, 43). These values are largely consistent with our observations. This consistency suggests that IVIM can reliably measure muscle properties in both healthy and pathological conditions, supporting its technical feasibility for detecting muscle changes.

In the present study, four RC muscles of the affected shoulder displayed decreased D* values compared to the contralateral shoulder at baseline. Since D* has been shown to correlate with microvascular blood velocity and microvessel density in previous studies, this may suggest that capillary depletion is more prominent in shoulders with RC disorders (22, 24). In chronically inflamed muscle, lower capillary densities and increased heterogeneity in capillary network spacing are commonly observed, which may lead to lower D* and fD*. Also, altered muscle physiology, including a decrease in muscle fiber force, ultrastructural abnormalities in contractile proteins, and the accumulation of interstitial and intracellular lipids due to chronic inflammation, may contribute to the reduction of the diffusivity of water molecules in skeletal muscle, which in turn affects lower ADC and D values. One previous study shared similar ideas, which found reduced supraspinatus muscle perfusion in the operated shoulder compared to the contralateral shoulder in patients undergoing arthroscopic rotator cuff repair (ARCR) using ultrasound (44).

While all IVIM-derived parameters showed reductions in shoulders with RC disorders, f values showed the opposite trend. Since f reflects the signal fraction of capillary blood flow in the entire water molecule diffusion pool within each voxel, it can be inferred that the obstruction of arterioles and venules is likely to account for the increase in f values. Narrowing of small arteries and veins at the capillary boundaries is commonly observed in pathological muscle tissue (25).

Additionally, four RC muscles display higher FF in the shoulders with shoulder pain. This may suggest that slight fat deposition occurs in the early pathological stage of the shoulder. For imaging values that differentiate degeneration from normal muscle, IVIM-derived D* appears to be more effective, which indicates that the capillary depletion is more prominent than FI in the early stages of shoulder pathology.

Since inflammation creates an environment that accelerates degeneration, fat infiltration is an adverse consequence brought with chronic inflammation (45). Thus, IVIM may provide us with a forward-looking perspective to evaluate the early pathological change in RC muscles with RC disorders.

Physiotherapy exercise is known to restore shoulder function and reduce pain for patients with shoulder issues, and it can be easily implemented with patients following a self-management plan at home. Exercise has been demonstrated to reduce inflammation, delay cartilage and bone degeneration, change tendon, and muscle structure (46). Moreover, combined with pharmacological AMPK activation, exercise could improve muscle function and regeneration (47). Three studies evaluated muscle perfusion changes in rotator cuff muscles with IVIM in healthy individuals after specific movement, while they mainly focused on the local muscle flow recruitment during a specific movement, such as a lift-off test (28, 29, 43). In the present study, we observed increased capillary flow reflected by D* in four muscle groups after exercise, especially in SSP. Notably, a weak but significant correlation was identified between D* changes in the SSP muscle and improvements in Constant and ASES clinical scores.

The specificity of this correlation to the D* parameter may be attributed to its physiological interpretation. Unlike other IVIM parameters, D* is considered particularly sensitive to the velocity and patterns of capillary blood flow, reflecting perfusion efficiency rather than volume. This suggests that functional improvements experienced by patients may be more closely associated with enhanced capillary flow dynamics than with changes in blood volume (perfusion fraction, f) or tissue cellularity (diffusion coefficient, D). The supraspinatus muscle's particular vulnerability to mechanical compression and its substantial load-bearing role during shoulder abduction may explain why perfusion changes in this muscle specifically correlated with clinical improvement. These mechanical characteristics likely make the SSP more susceptible to ischemic stress and subsequently more responsive to exercise-induced perfusion recovery. Although the correlation coefficients were modest, this finding aligns with existing literature; for instance, Klausen et al. documented a 20% increase in capillary density following long-term exercise training (48), supporting the biological plausibility of our observations. The moderate correlation strength likely reflects the multifactorial nature of clinical improvement, in which perfusion changes represent only one contributing element. These preliminary results highlight the potential of D* as a sensitive biomarker for monitoring rehabilitation and warrant further investigation in larger-scale studies to confirm and refine its clinical value.

Since exercise could transfer water from the vasculature to the intracellular (myofiber) and extracellular (endomysium) spaces, we also reported the increase of ADC and D values in most RC muscles (33). The decrease of f values in SSP and ISP suggests exercise helps alleviate obstruction of arterioles and venous, possibly due to the increased exercise load on these two muscle groups. Also, the decrease of f is associated with the improvement of VAS in SSP, which turns the subjective pain score into an objective measure to help clinicians better assess the patient. However, given the exploratory nature of this finding and the multifactorial nature of pain perception, it should be interpreted with caution and validated in larger cohorts. Unlike the improvement of capillary flow, no significant change in FF may indicate that fat infiltration, if present, requires longer duration to resolve. Or maybe this may suggest that the FI degeneration of RC muscles maybe irreversible (49, 50).

Moreover, along with the improvement of imaging parameters, the clinical scores of the patient also improved. Due to the relatively short follow-up period, the observed improvement in clinical outcomes may not solely be attributed to the rehabilitation exercises. Other factors, such as reduced workload on the affected shoulder and patient mood improvements, could also have contributed to the results. Nevertheless, our findings provide some evidence suggesting that the rehabilitation exercises we designed may effectively improve the functional outcomes of patients with shoulder disorders.

The findings of this study have several potential clinical applications. The IVIM-derived parameter D* could serve as a sensitive, quantitative biomarker for detecting early microvascular alterations in RC muscles, preceding overt fat infiltration. This could improve early diagnosis and help stratify patients for targeted rehabilitation programs. Furthermore, this technique provides a non-invasive means to objectively monitor patient response to physiotherapy, complementing traditional subjective scores. While our cohort consisted of patients with unilateral shoulder pain, the methodology is generalizable to other musculoskeletal conditions. For widespread clinical adoption, further validation in larger, multi-center cohorts and increased accessibility of IVIM/FACT sequences on commercial MRI scanners are necessary prerequisites.

There were some limitations to this study. First, this study has a small sample size. A formal *a priori* sample size calculation was not performed, as this was an exploratory study applying novel IVIM and FACT sequences to a new clinical context, and preliminary data for estimating effect sizes were lacking. Although our *post hoc* analysis confirmed that the achieved sample size provided sufficient statistical power (>80%) for detecting changes in our primary outcome measures (e.g., fD*, D* and FF), the power remained suboptimal for some secondary parameters with smaller effect sizes (e.g., f, D and ADC). Therefore, the findings related to these specific parameters should be interpreted as preliminary and generative of hypotheses for future validation in larger, adequately powered cohorts. Second, the follow-up time is limited to the early stage after physiotherapy exercise; more follow-up time points are needed to evaluate the muscle condition after exercise. Third, a few patients showed poor adherence to the exercise regimen. Strong monitoring measures, such as the implementation of video surveillance, should be incorporated to oversee patient behavior and ensure their well-being. Future studies should include both exercise, non-exercise groups and stratify patients based on severity to further explore the effect of exercise on improving the condition of the rotator cuff muscles.

## Conclusion

Capillary flow reduction reflected by IVIM-derived D* is more prominent than FI reflected by FF-derived FF in the early stages of shoulder pathology in patients with shoulder pain. Also, IVIM parameters, particularly D* may assist clinicians in more accurately monitoring patients' conditions post-rehabilitation.

## Data Availability

The raw data supporting the conclusions of this article will be made available by the authors, without undue reservation.
