# Supplementary material for: Evaluation of IVIM and FACT imaging for early detection of rotator cuff muscle degeneration and physiotherapy exercise effects
Source: Front Sports Act Living. 2025 Oct 30;7:1688162. doi: 10.3389/fspor.2025.1688162 (PMC12611895; doi:10.3389/fspor.2025.1688162)
Supplement: Supplementary file 1 [file Table1.docx]

| **Table S1. Details of the exercise rehabilitation movement.** | | | |
| --- | --- | --- | --- |
| **No.** | **Action Picture** | **Detailed explanation** | **Reference** |
| 1 | 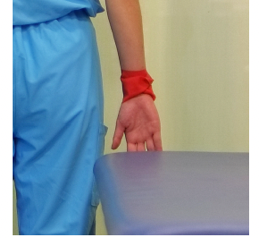 | **Low row：**  The low row (LR)is an exercise that emphasizes scapular external rotation and posterior tilt.  Each subject stood in front of an immovable surface. Subjects placed their hand on the anterior edge of the surface with palm facing posteriorly. Subjects were instructed to extend their trunk and push their hand maximally against the surface in the direction of shoulder extension and instructed to retract and depress the scapula; the isometric contraction was performed for 5 seconds.  Our setup: Perform 8 sets, each lasting 5 seconds. | Electromyographic Analysis of Specific Exercises for Scapular Control in Early Phases of Shoulder Rehabilitation |
| 2 | 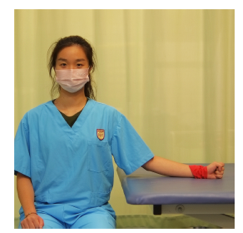 | **Inferior glide exercise：**  The inferior glide (IG) is an isometric exercise that emphasizes humeral head depression and scapular retraction.  Each subject was placed in an upright sitting position with the test arm abducted to 90° with fist clenched on a firm supportive surface. Each subject was instructed to apply pressure with his or her fist in the direction of arm adduction and instructed to inferiorly depress their scapula and hold this position for 5 seconds.  Our setup: Perform 8 sets, each lasting 5 seconds. |  |
| 3 | 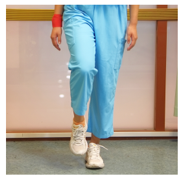 | **One-leg stance:**  A one-leg stability series, which assesses control of the trunk over the leg.  Our setup: Alternate between left and right for 1 set, perform 8 sets, each lasting 5 seconds. | Houlder Rehabilitation Strategies, Guidelines and Practice |
| 4 | 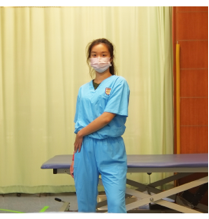 | **Hip extension: Trunk rotation**  Our setup: Perform 8 sets, each lasting 5 seconds. |  |
| 5 | 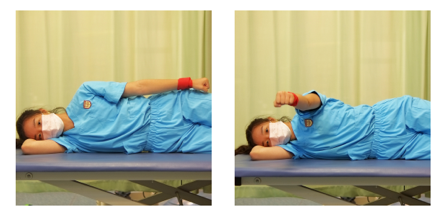 | **side-lying forward flexion:**  Subject in side-lying position, shoulder in neutral position; subject performs forward side-lying position flexion in a horizontal plane to 135°。  Our setup: No dumbbell weight, perform 8 sets, each lasting 5 seconds. | Rehabilitation of Scapular Muscle Balance Which Exercises to Prescribe? |
| 6 | 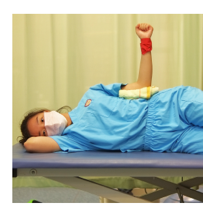 | s**ide-lying external rotation:**  Subject side-lying with the shoulder in neutral position and the elbow flexed 90°; subject performs external rotation of the shoulder (with towel between trunk and elbow to avoid compensatory movements)  Our setup: No dumbbell weight, perform 8 sets, each lasting 5 seconds. |  |
| 7 | 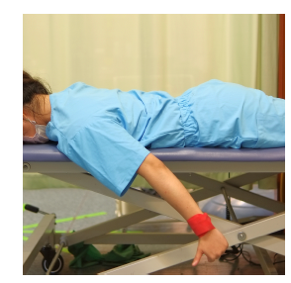 | **horizontal abduction with external rotation:**  Subject prone with the shoulders resting in 45° forward flexion; subject performs horizontal abduction to horizontal position  Our setup: No dumbbell weight, perform 8 sets, each lasting 5 seconds. |  |
| 8 | 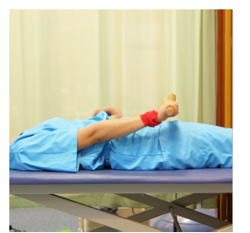 | **Active-Assisted and Active ROM:**  This includes four active range of motion exercises with the use of a stick: forward flexion, abduction, internal rotation, and external rotation with the stick.  Our setup: Perform 8 sets, holding the end position for 5 seconds each time. | Pre-Operative Scapular Rehabilitation for Arthroscopic Repair of Traumatic Rotator Cuff Tear: Results of a Randomized Clinical Trial |
| **9** | 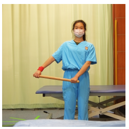 |  |  |
| 10 | 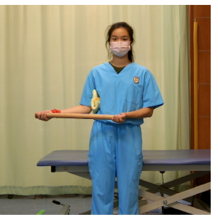 |  |  |
| 11 | 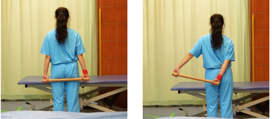 |  |  |
| 12 | 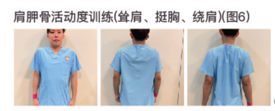 | **Shoulder shrug:**  Our setup: Perform 8 sets. |  |
| **13** | **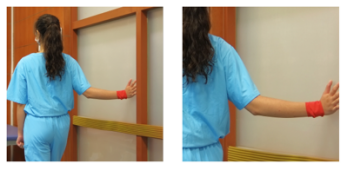** | **Corner stretch:**  Our setup: Alternate between left and right once each, holding each position for 30 seconds. |  |
